# Youth as contested sites of culture: The intergenerational acculturation gap amongst new migrant communities—Parental and young adult perspectives

**DOI:** 10.1371/journal.pone.0170700

**Published:** 2017-02-07

**Authors:** Andre M. N. Renzaho, Nidhi Dhingra, Nichole Georgeou

**Affiliations:** Humanitarian and Development Research Initiative, School of Social Sciences and Psychology, Western Sydney University, Sydney, New South Wales, Australia; Royal Children's Hospital, AUSTRALIA

## Abstract

**Background:**

Immigration often results in changes in family dynamics, and within this process of dynamic relational adjustment youth can be conceptualised as contested sites of culture and associated intergenerational conflicts. This paper considers the experiences of migrant youth in Greater Western Sydney, New South Wales, Australia using conflict as a useful lens through which to view issues of migrant youth identity and their sense of social connectedness, belonging, and agency. The aim of this study was twofold: 1) to explore how migrant youth cope with acculturative stress and intergenerational conflicts, and 2) to better understand the systemic and family-related factors that facilitate positive settlement experiences for migrant youth.

**Methods:**

A total of 14 focus group discussions, comprising 164 people, were carried out in Greater Western Sydney, New South Wales, Australia. These focus groups targeted newly arrived migrant parents and young adults (aged 18–24) of African, Burmese, Nepalese, Indian, Afghani, Bangladeshi and Iraqi backgrounds. Each focus group was 1.5 hours in duration and was conducted by a team of three people (an experienced facilitator, an accredited interpreter/bilingual worker, and a note taker). Data were collected using a standard interview schedule, and an accredited interpreter/bilingual worker asked the questions in the appropriate language and translated participant responses into English.

**Results:**

The findings highlight how youth in new migrant families become contested sites of culture as they try to balance integration into the new culture while maintaining their originating country’s cultural values. Two themes and four subthemes emerged from the analysis: Intergenerational acculturation gap (loss of family capital and intergenerational conflicts); and factors that successfully protected positive family values while still allowing young people to integrate (the legal system that disarm authoritarian parenting practices and family rules; and parental use of children’s increased knowledge of the new environment to navigate their new environment). Migrant families conceptualised family capital as the social solidarity, influence, and control governing obligations and expectations, intergenerational knowledge transmission and information flow, social norms, and cultural identity. The loss of family capital was characterised by children’s refusal to associate with or meet family members, preferring to be alone in their rooms and private space. Migrant youth find themselves caught between and negotiating two cultures, with unwanted negative consequences at the family level in the form of intergenerational conflicts. The new found freedom among children and their rapid transition into the Australian society gives children an increased sense of agency, which in turn threatens parental authority, allowing children to exercise three forms of power: increased assertiveness due to legal protection of children against any corporal punishment; and English language fluency and greater understanding of the functioning of Australian social institutions.

**Conclusion:**

Our findings suggest the need for an inter-generational approach to healthy family dynamics within migrant communities when dealing with youth negotiating the complexity and sensitivity of forging their cultural identity.

## Introduction

Migration policies in most western countries have been subject of significant policy reforms. For example, in 1973 Australia adopted a non-discriminatory and non-assimilationist migration policy that enabled anyone to migrate to Australia, regardless of race, colour, gender, ethnic origin, religion, or nationality. [1, 2] This ended the previous “White Australia” Policy, which from 1901 had shaped the composition of the Australian population through mass migration programs for British and European immigrants. This shift from assimilation towards an official policy of multiculturalism was in part a recognition that, despite migrants facing challenges when settling in Australia, they remained attached to their cultural identity[2]. The shift in migration policy has seen Australia’s population become more ethnically diverse, rising from 9.8% overseas-born in 1948, to 19.8% by 1970, and to 27.7% by 2013. [3]. Similar policy reforms and associated demographic transformation of the migrant-receiving populations have been documented in other western countries[4–6]

Migration is a complex phenomenon, which involves people coming to western countries through various migration streams, including skilled, family, and special eligibility categories, as well as refugee and humanitarian programs [5, 7, 8]. Western governments have emphasised that most migrants must have skills or the professional expertise to meet the needs of their labour force, so as to directly benefit their economy. Therefore, western countries use a “hybrid” model of skilled migration that selects migrants using both a supply-driven model (i.e. using points-tests to screen in favour of prospective skilled migrants with desirable characteristics who put themselves forward for consideration) and demand-driven model (i.e. employer-sponsored skilled migrants who are likely to contribute positively to the receiving economy)[9].

Whilst settlement and integration are challenges for most migrants, such challenges are likely to be more pronounced among the refugee and humanitarian migration stream due to their socio-economic disadvantage. Over the last four decades, many western governments strengthened their policies on multiculturalism and social cohesion in their efforts to address the impact of increased cultural diversity within communities[10]. The notion of living with difference that characterises multicultural societies can leave migrant youth in limbo. For example, the acculturation theory, which permits the examination of cultural and psychological changes that occur when two cultures come into contact with each other, [11, 12] acknowledges that migrants experience varying inequalities. These include: difficulties establishing social networks, finding accommodation or employment, learning English, and looking after their general health. However, the level of inequality differs according to the degree of cultural transition and generation.[13–18] For migrant youth, the bi-dimensional acculturation model recognises two cultural orientations—the home culture and the host culture—and identifies four cultural orientations: (1) Traditional or separation (keeps loyalty to traditional culture and does not recognise the host/dominant culture); (2) Assimilation (rejects traditional culture and fully embraces the host/dominant culture); (3) Integration or bicultural orientation (retains cultural identity at the same time moving to join the dominant society); and (4) Marginalisation (rejects traditional culture and fails to connect with the host/dominant culture by exclusion or withdrawal.[13, 19, 20] In this sense, regardless of their length of stay in their host country, migrant youth experience issues related to cultural identity, social belonging and cultural attachment due to the more rapid pace of their acculturation compared to their parents.

During adolescence children go through a phase in which they attempt to reconcile their own desires and needs with the wishes of their parents[21], including wanting privacy, negotiating parental monitoring and control (e.g., attention, tracking, and structuring contexts) and parent—child relationship (e.g., trust), and having agency and pushing back parental behavior management (e.g., negotiation, problem-solving, or limit-setting) and demand cognitions concerning parent-child interactions (e.g. parents’ beliefs that they should decide what the children want and need) [22–25]. However, there are some cultural differences in parenting behaviors and practices. In most western countries parenting behaviours and practices are conceptualized within the individualistic cultures[26]. The emphasis is on child’s autonomy, independence and self-reliance; individual achievements; individual responsibility; equality of individuals; liberty from inference of others; and conformity to firm individual boundaries[27]. Parents adopt a more authoritative approach, striking a balance between high parental demandingness and the recognition of children’s autonomy and rights[27]. Parents treat children with respect while instilling in them a strong inner discipline and encouraging open communication and children’s expression of their point of view [28–30]. Migrants from Africa, the Middle East, and Asia relocating to western countries come from collectivist cultures [26]. In collectivist cultures, the emphasis is on interdependence and conformity to collective responsibility within the family, indisputable obedience and respect for authority, especially respect for the family hierarchy and elders[27]. Parents adopt an authoritarian parenting style characterized by exerting control through power, coercion, and punishment. Children’s input into parent-child communications and decision making that might impact of their future plans is devalued. Parents expect children to adhere to family rules and regulations; and are the ultimate decision makers and expect children to conform to parental demands without dispute [28–30].

Migrants from collectivistic to individualistic countries find it difficult to negotiate the acculturation process [31]. The cultural distance between the pre-migration collectivist cultural orientation and the post-migration individualistic cultural values not only leads to acculturative stress but also to parenting stress. The acculturation stress in characterized by somatic, psychological, and social difficulties that accompany acculturation processes resulting in physical and mental maladaptation[32]. The acculturative stress affects negatively the ways in which parents interact with their children and parent-child interaction difficulties and family conflicts are further aggravated by time demands and work stress and work-family conflict. Children pick up the Australian cultural norms and understand the legal system quicker than their parents and use acquired knowledge to disarm authoritarian parenting practices and family rules. The unfamiliar power of state intervention to intervene in family matters for disciplinarian practices is an omnipresent threat, which appears to have far reaching effects on the family functioning [24, 25, 33]

Therefore, the intergenerational discrepancy theory, also known as acculturative family distancing, conceptualises the clash of values and expectations between migrant parents and their children due to the differing pace of acculturation, resulting in increased family conflicts, parent-child alienation and maladjustment of children.[34–36]. The intra-familial and interethnic dynamics that migrant youth are exposed to are characterised by a breakdown of communication (both verbal and nonverbal) and poor parent-child relations which together form their journey towards cultural identity formation.[36] In the Australian context, as migrant youth push hard to be part of, and to be accepted by, the wider community, they detach themselves from their culture of origin, [16, 18, 33] and in so doing, face resistance from their parents. The more they are made to feel they do not belong, the higher the likelihood they will disengage from Australian society. Studies by Mansouri and Johns, [37] as well as by Cassity, [38] have observed that migrant youth develop multiple expectations in their attempt to reconcile the difference between their aspirations and those of their parents, as well as their hopes for the future in Australia, with families and friends in their home country. They conclude that intergenerational differences become a lens through which to view issues of migrant youth identity and their social connectedness, belonging, and agency. Therefore, the aim of this study was twofold: (1) to explore how migrant youth cope with acculturative stress and intergenerational conflicts, and (2) to better understand the systemic and family-related factors that facilitate positive settlement experiences for migrant youth.

## Methods

### Study design, setting and governance

This exploratory qualitative research was carried out in Greater Western Sydney, New South Wales, Australia, a region that includes the northwest, southwest, central west and western sub-regions of greater metropolitan Sydney. Greater Western Sydney covers an area of approximately 8,941 km^2^ and has a population of 1,923,698 usual residents[39], and is the fastest growing and most ethnically diverse region in Australia. The qualitative approach was the most appropriate research design for this study. That is, the issues of migrant youth identity and their sense of social connectedness, belonging, and agency are often poorly understood and under-documented[10, 40]. Using a qualitative design allowed the research team to understand the context and environment in which migrant youth negotiate their identity and sense of social connectedness, belonging, and agency, to further explore how they put meanings to these concepts, and to provide meaningful data that will inform hypotheses formulation for further testing in quantitative studies. In order to cover various aspects of acculturation, the study focused on newly arrived migrants of African, Burmese, Nepalese, Indian, Afghani, Bangladeshi and Iraqi backgrounds on how their families, in particular their children, had integrated into Australian society. The study included migrants from all migration streams, including refugee and humanitarian entrants, family reunion, and skilled migration. A Migrant Review Panel was established in March 2015 as a *de facto* community advisory committee comprising 22 community leaders and lay representatives from migrant communities. This brought guidance and insights to the research process in addition to contributing to all aspects of the project, including participants’ recruitment, the development of the interview guide, feedback on the study findings, and feeding the results back to the community. Panel members were either invited by the research team or nominated by their respective communities.

### Recruitment and data collection

Study participants were recruited using purposeful sampling. Three criteria were employed in the selection of participants: (1) geographical (according to their re-settlement location), (2) by country of origin, and (3) by length of stay in Australia. They were recruited with the assistance of bi-cultural community workers through the Migrant Review Panel, SydWest Multicultural, Health Services, and community-based associations. Data were collected using focus group discussions (FGD). The International Labour Organization defines a youth as any individual in the 15–24 year age bracket[41]. In our study we excluded participants younger than 18 years and focused on parents and youth aged 18–24 years for many reasons. Firstly, focus groups were the method of choice due to the influence of the social theories of collectivism and oral literacy on the methodology as collectivism and oral literacy are embedded in the social and cultural life migrants from low and middle income countries. Collectivist elders (e.g. parents) are perceived to have a lot of authority, and their main role is to transfer knowledge to the children. Therefore, parents were reluctant to allow for children younger than 18 years to participate in the focus group discussions alone, and where consent was obtained, parents insisted on being present. Having focus group discussions with children younger than 18 years meant that parents had to be present and they would speak on behalf of children. Secondly, in contrast to individualist ideals, collectivism shapes knowledge exchange and promotes decision-making as a process that is heavily influenced by wider family and social networks[42]. Thirdly, oral literacy is a major feature of managing and transmitting knowledge[43] that draws on relationships between people, allowing them to participate in a shared meaning-making in distinctive ways[44]. Sociability, then, was an important construct in the selection of focus groups as the interaction in group discussion is known to push the boundaries of what can be gained from one-on-one interviews[45]. Finally, the focus group discussions were drawn from existing networks. In order to enhance contribution and manage issues of community dynamics such as young people being perceived as having betrayed their communities by discussing sensitive issues outside the collectivist communication hierarchies, focus group discussions included a mix of youth and parents. Focus group discussions were organised by country of birth and language spoken at home. Experienced bilingual facilitators moderated the focus group discussions to ensure young people spoke freely while at the same time ensuring youth showed respect to elders and thereby avoiding the likelihood of suppressing younger participants’ views in a multi-generational forum.

Given that the study participants had a varying degree of exposure to the Australian environment, in order to account for the differing level of acculturation, focus groups were comprised of participants who had been in Australia for different lengths of time, from 6 months to 5 years. Prior to data collection, researchers and community bilingual workers met with the community members and explained to them the study’s objectives and its intended outcomes using a plain language statement. Participants were then invited to take part in the study, stressing that any involvement was voluntary. Written consent was obtained from all participants except for those who had low text based literacy where verbal consent was audio-recorded. All written consents are stored in a safe place only accessible by the lead researcher and the research assistant. All audio-recorded consents are stored on a pass-word protected computer only accessible by the lead researcher and the research assistant. Participants who took part in the study received a $25 supermarket voucher as reimbursement for their time and any associated travel costs. The study procedures and protocol were approved by the Western Sydney University Human Research Ethics Committee (H11213).

The focus group discussions were facilitated using approaches we have successfully used in previous studies.[16–18, 33] Each FGD was led by three people: an experienced facilitator, an interpreter/bilingual worker, and a note taker. Data were collected using a standard interview schedule, made of open-ended questions formulated using a review of the existing evidence and acculturation theory, with input from Migrant Review Panel members. The facilitator asked questions from the interview schedule (which was translated by the bilingual worker) and managed the conduct of the group discussion ensuring that quieter participants had the opportunity to speak. The interpreter/bilingual worker asked the questions in the appropriate language and translated participant responses into English. Interpreters/bilingual workers had the opportunity to ask probing questions in order to clarify responses and facilitate the flow of the discussion. The note taker made written notes about key concepts and identified points for later discussion and clarification. These notes assisted with subsequent data coding. Data collection and preliminary analyses were conducted iteratively, with one FGD informing subsequent FGDs until it was agreed by researchers (ND, AR, and Migrant Review Panel) that no new themes were emerging. The focus groups were held in a community setting (e.g. a community hall or settlement service centres) and lasted approximately 90 minutes. With the permission of participants, all focus group discussions were audio recorded and later transcribed verbatim for analysis by an experienced transcriber. Data analysis.

In order to increase the trustworthiness of our findings, a number of strategies were implemented [46]. We used iterative questioning during FGDs using probes to elicit detailed data to complement responses and/or to refer to matter previously raised by participants in order to establish clarity, synergy and contradictions in participants’ responses. The research team had frequent debriefing sessions with steering committee members to comment on emerging findings. Their inputs were paramount in revising our findings. The final findings were presented to services providers to illicit their reaction and final input as part of the process to finalise our thematic mapping and final report. Transcripts were manually coded by two members of the research team (ND & NG) and then reviewed by a third researcher (AR) to improve the rigour of the study.[47] The coding followed six discrete steps recommended by Braun and Clark.[48] The first step involved familiarization with the data, including reading and re-reading the transcripts in order to obtain a broad understanding of participants’ responses. The second step involved the generation of initial codes. The third centered on grouping codes into potential themes. In the fourth step, identified themes were reviewed and a thematic map of the analysis was generated. In the fifth step, themes were defined and named, and potential subthemes generated until saturation of themes with “thick” description of the data was reached and no new data emerged to support each theme.[49] The last step involved narrating the results, and supporting each theme with the participants’ voice. Illustrative exemplars were chosen to represent the richness of the data and theme representativeness, [50] and the original syntax used by FGD participants has been retained.

## Results

A total of 14 FGDs, comprising a total of 164 people, were carried out. The demographic characteristics of participants are shown in Table 1. Youth (18–24 years old) formed part of FGDs, however most participants were the adult parents of youth (13–17 years old). Two themes and four subthemes emerged from the analysis: Intergenerational acculturation gap (loss of family capital and intergenerational conflicts); and factors that successfully protected family values while still allowing young people to integrate (the legal system that disarm authoritarian parenting practices and family rules; and parental use of children’s increased knowledge of the new environment to navigate the new environment).

**Table 1:** Demographics characteristics of focus group participants, listed in the order conducted.

FGD No.	Background	No. of people per FGD	Age range (Years)	Median age of participants (Years)	Gender make up	Migration stream (refugee/humanitarian; family/partner; skilled)	Length of stay in Australia (> 5 yrs, < 5yrs)
1.	Afghanistan	10.0	58–77	66.5	100% M	refugee/humanitarian	more than 5
2.	Bangladesh	11.0	22–52	40.0	36.4% F 63.4% M	mixed visa status	mixed
3.	Iraq	10.0	28–69	55.5	10% F 90% M	refugee/humanitarian	less than 5
4.	Nepal	11.0	46–73	59.5	100% M	refugee/humanitarian	mixed
5.	Afghanistan	13.0	21–62	54.0	100% F	mixed visa status: refugee/humanitarian family/partner	mixed
6.	Nepal	15.0	21–59	47.0	100% F	refugee/humanitarian	mixed
7.	Bangladesh	13.0	24–47	40.0	23.1% F 76.9% M	mixed visa status	mixed
8.	African*	13.0	24–58	49.0	63.6% F 36.4%M	mixed visa status: refugee/humanitarian family/partner	mixed
9.	Burmese	13.0	23–80	38.0	61.5% F 38.5% M	mixed visa status: refugee/humanitarian family/partner	mixed
10.	Iraq	11.0	36–66	52.0	100% F	refugee/humanitarian	less than 5
11.	Iraq	6.0	20–29	23.0	100% F	refugee/humanitarian	mixed
12.	African*	7.0	31–54	35.0	14.3% F 85.7% M	mixed visa status: refugee/humanitarian family/partner	
13.	India**	14.0	18–65	43	64.3% F 35.7% M	mixed visa status	mixed
14.	India**	17.0	20–68	38.5	100% F	mixed visa status: refugee/humanitarian & family/partner	mixed

* African group participants were purposively recruited from across the continent for diversity and data richness

** Indian group was a mixed of migrants from India and Pakistan from existing groups of the community organisation

### Intergenerational acculturation gap

#### Loss of family capital

Migrant families conceptualised family capital as the social solidarity, influence, and control at the family level that governs obligations and expectations, intergenerational knowledge transmission and information flow, social norms, and cultural identity. Participants noted that the loss of family capital was characterised by impaired family-unit level attributes whereupon migration migrant children ‘no longer like to associate with their family’ and ‘prefer to separate from the rest of family members’. As parents observed:

“We are used to a family that we communicate, we do everything together. So firstly in here, your own child, they always want our privacy. You can’t just enter their room. Although you’re paying for their rent but you can just enter the house. And they choose to tell you what they want you to know.”

(African female, FGD 8)

Children do not want to meet their extended family, and do not respect or obey their parents; rather, they prefer to stay in their rooms citing ‘space’, and ‘privacy’ as reasons for keeping to themselves. Indeed, they want their own bedrooms. At the same time, young adults explained that part of the reluctance of young people to mix with family and community members was due to a social intergenerational divide that was of little interest to them. The following comment from a young adult highlights intergenerational difference in migrant identity, and the loosening of social connectedness to parents:

“Lots of times before we used to go together to see like friends, like I mean relatives and stuff, but now only mum and dad go and visit them…’cause even if we would go with them, there'd be no, not kids our age, there'd be like just the parents, and like why are we going there?”

(Iraqi young adult participant, FGD 11)

Parents’ lack of understanding about their children’s desire for privacy and social independence let to parental anxiety about the activities of their children when they were away from the family:

“It’s just a different world that you are in, you don’t know what your child is doing, completely. So at the end of the day the negative aspect of it is, maybe they be drinking out there, you don’t know. They may be smoking out there and you’re not aware. They are—yeah the alcohol is there, we don’t know. Because when they come before us they just come and go into their room, lock themselves up, and next morning they go. So because there is no—even you try, like the family dinner and everything.”

(African female, FGD 8)

Parents also acknowledged that children have more exposure to the host/dominant culture because at school they learn about the new culture, socialise with schoolmates, listen to English music, and adapt faster to their new cultural environment. A youth from Iraq describes the tensions that arise in families as children adapt to the host culture more quickly than their parents.

“…if they [children] come here young, they adapt to society, their parents don't want them to lose their culture 'cause they think, ‘This is what we want our kids to be.’ And like say, with physical punishment and all of that, they still think it's right. Well some people don't do it as much now, but they think that ‘Oh, our children are adapting in to this society and they're forgetting our culture’, and they don't want that. So there's always conflict between how kids are growing up here and they're forgetting their culture.”

(Iraqi young adult participant, FGD 11)

While attendance at school facilitates children’s integration in the new environment, parents, in contrast, do not have that social exposure or a social circle to facilitate their interaction with the new environment, so they continue to follow news in their home country and to hold on to their older cultural values. The different environment between school and home and associated acculturative stress means that children find themselves confused as to which values to hold on to, while parents try to adapt but fear that the children will lose their culture. Migrant youth find themselves caught between, and negotiating within, two cultures with unwanted consequences at the family level. When confronted with such a dilemma, migrant children tend to favour the host culture’s social and cultural norms to fit in. An Iraqi young adult participant articulated this tension:

“…I have to respect my dad and my mum, 'cause still I'm stuck in the culture as well, 'cause I came to Australia not too long ago. Sometimes I feel no, I can't put my parents in a situation they‘d be ashamed.”

(Iraqi young adult participant, FGD 11)

The difference in the pace of acculturation between children and parents leads to challenges related to parental expectations of their offspring. However, these are changes in traditional cultural values that do not sit well with parents, and adapting to Australian culture was linked to a loss of traditional values as they adopted “Australian values”. A participant noted:

“A lot of the time they spend with them in schools, six or seven hours, you think that in 24 hours, six, seven hours, so most of the time they spend of the day, and their values are changing.”

(Bangladeshi male, FGD 7)

We found that parents use a number of restrictive strategies to deal with the difficulties associated with the double cultural identity youth are confronted with post migration. Firstly, parents expressed a fear of their children disconnecting from the traditional culture and values of their family and sought to implement rigid family rules to reinforce traditional values.

“We wanted to keep our values and culture but we are worried that our kids won’t follow that culture or that value, they will adapt to the new culture.”

(Afghani female, FGD 5)

Therefore, parental fear and distrust of the host/dominant culture led to tight parental control, close scrutiny of children’s behaviour with restrictions, culture clash around physical punishment or talking loudly. One parent described her controlling behaviour:

“I call him every hour to ask what he’s doing. He says, mum you’re too boring. I say, ‘Whatever you want to say’, but I guarding him, you know. And taking him out of trouble. Because the children here, when they come here, they follow the system here. They want to be out there doing different things.”

(African female, FGD 8)

Secondly, children learn quickly that, in Australia, it is okay to have a girlfriend, to sleepover or invite a friend for a sleepover, and to date or marry outside their communities (values that do not sit well with their traditional cultural norms). A parent described the concerns that these new cultural activities raise for them:

“… she just tells you ‘my friend at school invited me for a sleepover’. I will not allow her to go before I learn exactly where she’s going. I will make sure that I know exactly if she’s going to her friend’s house and if also the parents will accept her.”

(African male, FGD 12)

Parents also acknowledged that they put pressure on their children to stay within their own community:

“There is a fear, the community is very small, and if my daughter or son is seen with another guy… Yeah, outside the culture, ‘my daughter has a boyfriend’, and my neighbour knows it, the whole community will know it. Big fight.”

(Bangladeshi male, FGD 7)

Finally, the fearful protectiveness of parents placed pressure on their children. A young Iraqi adult described his experience:

“If you stay out 'till 12 o'clock… my parents will call, like every five minute, ‘Where are you?’, “Where have you been?’, ‘Why are you not home?’”

(Iraqi young adult participant, FGD 11)

However, it is interesting to note that participants identified different restrictions for sons and daughters, and the children themselves could see some parental actions as an invasion of their privacy. Young adults were also aware of the gendered restrictions on their behaviour, and noted that this was another point of conflict between them and their parents:

“Before they would let my brother go out, and he was younger than me too, they would let him go out late or would let him date, but not me. And I was older. And I'd always argue over that, would always have clashes because of girls can't really protect themselves if they go out late, or whatever, they're just like… ‘Not only they can't protect themselves, but there's also the reputation side of things.’ Like if you go out…”

(Iraqi young adult participant, FGD 11)

#### Intergenerational conflicts

The new found freedom among children and their rapid transition into the Australian society gives them an increased sense of agency, which in turn threatens parental authority, leading to compromised parent-child relationships and subsequent intergenerational conflicts. Participants noted that children think their parents are too old-fashioned, whereas parents think their children have been negatively affected by the new environment. Therefore, intergenerational conflicts identified by participants centred on *poor communication* between parents and children, an outcome of many factors including: lifestyle related changes (e.g. no time to communicate or resolve issues due to everyone being busy); language barriers (e.g. children refuse to interact with parents in their mother tongue as they find it alienating while parents are unable to understand or speak English); disruption of family dynamics (e.g. increase in family conflict due to acculturation gap); and parenting ways (e.g. putting pressure on children to comply with parental demands or restricting children’s liberty and decision making). Indeed one parent described her children’s adoption of the host/dominant culture as “invasion”:

“Regular communication with the children is the only way to keep them… from that invasion. I must say invasion.”

(Pakistani female, FGD 13)

Parents also said they felt alienated from their children as spaces for communication and connection that were linked to traditional gender roles were eroded:

“They are so much busy into their personal own type of life that they won’t get time to come and check with me and talk with me or go to the kitchen or help me with the kitchen, so she’s always grooming her hair or being on mobile for so long, and sometimes I get so nervous that I just want to go and cut the hair…”

(An Afghani mother, FGD 5)

Participants noted that the net effect of poor communication between parents and children was compromised family cohesion and increased stress in families. A young Iraqi adult observed:

“… that family unit is breaking, because basically parents and kids are never understanding each other, … kids think ‘oh, my parents are old fashioned’, and parents are thinking, ‘oh my kids are getting corrupted’.”

(Iraqi young adult participant, FGD 11)

Parents described feeling inferior to their children when they realised that their children were better able to communicate in English than them. They noted that this situation posed a challenge to their parental authority, as well as their understanding of the parental role as transmitters of intergenerational knowledge:

“Easy to our kids at school, they learn the language so quick and difficult for us as middle aged parents. Sometimes we're embarrassed in front of our kids. I have a child at primary school and he told me about whales, and my son, I wasn't able to communicate at the same level, and that's really embarrassing me as a Dad that I can't communicate as naturally. In fact, our kids are able to communicate and teach us and that puts more pressure on us and our psychology and it will affect us.”

(Iraqi male, FGD 3)

Parents described the dilemmas and tensions they experienced as they sought to guide their children who moved between two cultures, each with different expectations:

“My daughter’s just going to school… she wants to have Christmas, and I said, ‘We’re Muslim, we don’t follow Christmas’. She said, ‘I love Christmas’, she loves Santa, and she wants to have a photo with Santa, and I said, ‘No, you’re Muslim, you shouldn’t be doing this.’ So doing Christmas in all lights, you know, everyone’s got lights in our neighbours. These are the challenges that we are facing. Obviously we’re trying to consult with her, this is our religion, we shouldn’t be doing it. She loves Peppa Pig, because she wants to buy a pillow or whatever, that has Peppa Pig, but that’s fine if you want to buy it, but I don’t like this…”

(Bangladeshi male, FGD 7)

Young adults also discussed their experience of conflict as they described tension around letting go of traditional cultural markers as they engaged in Australian society. A young Pakistani commented:

“My mum came in 2013. Before that I was alone. When my mum came here, she was like, ‘You’re not doing that.’ I was like, ‘Mum please, I won’t be able to wear burqa in my workplace. I can’t’. And she was like, ‘Why you not wearing scarf in your school?’ I was like, ‘I can’t really. I can’t do it. I am a totally international student. I won’t be able to do that.’”

(Pakistani young adult participant, FGD 14)

### Factors that successfully protected positive family values while still allowing young people to integrate

#### The legal system that disarm authoritarian parenting practices and family rules

The legal system that disarm authoritarian parenting practices and family rules and associated increased power of children relative to their parents featured predominantly in all focus group discussions. Participants believed that, upon migration, there is a change in power dynamics within the family, and parents lose control over children who develop power they did not have prior to migration. One form of increased power of children derives from Australian law, which protects children against any form of corporal punishment or parental neglect. Children pick up the Australian cultural norms and understand the legal system quicker than their parents and use the acquired knowledge to disarm authoritarian parenting practices and family rules. The unfamiliar power of state intervention to intervene in family matters for disciplinarian practices is an omnipresent threat, which appears to have far reaching effects on the family functioning. A grandmother described the dilemma that Australian laws on smacking children created for her when disciplining her grandson:

“To my grandson, who kept playing with the clock, I say to him, ‘If you do that again I will spank you’, and he said, ‘Grandma I will call the police.’ A two and a half years old child, who taught him about police?”

(African grandmother, FGD 8)

#### Parental use of children’s increased knowledge of the new environment

Another sense of power held by children is due to their superior language skills and greater understanding of the functioning of social institutions. This results in parents becoming dependent on their children to navigate the new system, resulting in a loss of control over their children. Parental use children’s increased knowledge of the new environment to maintain effective family functioning and are forced to negotiate with their children

“… They are educated, they know everything, so sometimes we have to depend on them. We give them the power in the family to go for marketing or doing anything. So whenever they do some mistakes, we just advise them. And after that they are being given all the power… they know that we are depending on them.”

(Nepalese male, FGD 4)

Participants emphasised that as their children guide and help them to use public transport and to complete daily chores and activities outside the home, their children have gained authority. As one participant summed up:

“Because our children catch the language very good they… the authority become on their hands.”

(Iraqi female, FGD 10)

However, as one parent noted, children don’t always help because “they get tired and bored of doing so”:

“All the time I depend on my children, but then my children become boring of me, and they told me ‘oh, stop mum, you have to learn’ Yeah.”

(Iraqi female, FGD 10)

Young adults were also aware of the tensions created by the multiple factors that provided children with increased power within the family:

“Our parents feel…[The system] turns them against their parents. And they feel like that we have no control over our kids once they leave to go to school, they're no longer our kids, and you can't do… like we can't raise them the way we want too.”

(Iraqi young adult participant, FGD 11)

## Discussion

The study explored how migrant youth cope with acculturative stress and intergenerational conflicts. It sought to understand the systemic and family-related factors that facilitate positive settlement experiences for migrant youth. The study identified two key issues related to the intergenerational acculturation gap: loss of family capital and intergenerational conflicts. The loss of family capital was characterised by children not wanting to interact with family members and negotiating two cultural identities. Research has shown that migration-related transition requires a massive revaluation of migrant capital (e.g. networks, cultural identities, and coping strategies.[51, 52] Previous studies have shown that, while migrant children upgrade the status of their family through education and integration within the mainstream society, they equally experience the same problems as their parents, but they also tend to get more help, or be helped sooner, than their parents.[53]

Despite the help they receive, migrant children experience more pressures from both their own families and from mainstream society to do well, as well as complying with expected home and host social and cultural norms, which sometime can be incompatible. We found that when confronted with such a choice, migrant children will tend to favour the host culture’s social and cultural norms to fit in, which is consistent with the literature.[16–18]. This is because values and ideals emphasised by parents in socialising their children attract negative reactions and resistance from children, leading to the alienation of parents from their children, and isolation of children at the family level. Migrant children’s demand for greater freedom increases as traditional social control measures weaken, requiring some fundamental changes in important cultural values and ideals related to respect for age and parental authority.[54] It takes time for traditional social measures among migrants to weaken, meaning that in the early phase of settlement, intergenerational cultural misunderstandings disrupt the social solidarity, influence, and control at the family level, and differing sets of obligations and expectations are adopted, resulting in the loss of intergenerational knowledge transmission and information flow. Children’s quest to fit into the mainstream society is characterised by adaptation to the new school and culture, experiencing associated racism or anti-immigration sentiments, making new friendships, and dealing with cultural loss and loneliness.[51, 55, 56] For migrant youth, relationships with peers might be an important and significant social resource, but they also pose a major issue.[56] It is then hypothesised that the cultural capital that is valued within the host society may not be easily accessible to newly arrived migrant people in the short term, [55, 56] leading to acculturative stress and associated family conflicts.

The study has also found intergenerational conflicts were characterised by an increased sense of agency among children, which challenged parental authority. These findings are consistent with past research.[13–18] It is well documented that members of migrant families with limited English encounter difficulties interacting with the mainstream society, and that parents experience difficulties in supporting children’s social and cultural transition.[33, 57] Migrant children’s social and cultural transition translates into their ability to take advantage of available resources to ease their integration journey into the host society.[58] They incorporate cultural capital including the use of language, social competence, and knowledge about the functioning of social institutions.[56] In doing so, some features of the family functioning are negatively affected, leading to conflicts. Affected dimensions of the family's cultural capital include verbal interaction, affective parent-child relationships, extent of discipline and control, parents’ expectations of their children's achievements, and parents' cultural beliefs and attributions.[14–18, 33, 59] The inter-generational conflicts may be exacerbated by migrant parents’ limited English language skills and expectations of strong social and family ties, which to some extent prevent them from fully participating in the wider community.[60] Some authors have observed that strong family or community networks can lead to negative social capital, with strong social networks established among co-ethnics isolating many migrants and locking them into specific “ethnic” niches.[51, 60] This further exacerbates intergenerational conflicts as ethnic niches may tend to enforce the control of children’s activities through discipline and an ethos of hard work in schools, which may be against children’s aspirations.[56]

The study also found factors that successfully protect positive family values while still allowing young people to integrate. Children have increased power under the law, due to their superior language skills and greater understanding of the functioning of the social institutions. In our previous studies [16, 17] we have demonstrated that, in their new environment, migrant children quickly become familiar with state laws protecting the rights of a child. They are able to disarm authoritarian parenting practices by the threat that parental violation of state laws will lead to family separation. Parents’ fear of state authority to remove children manifests itself in the perceived conflict between the need to discipline their children and Australia’s “anti smacking” laws, which are backed by police powers of arrest.[17] Migrants’ children appear to exercise limited agency in using this belief to challenge control and authority within the family.[60, 61] The unfamiliar power of state intervention to separate a family for strict discipline practices is not only an omnipresent threat, it appears to have far reaching effects on family functioning and dynamics.[17]

We also found a greater parental use of children’s increased knowledge of the new environment to navigate the new environment. Children of migrant parents from non-English speaking backgrounds learn English quickly through schools, and by interacting with their peers. This rapid English acquisition allows them to become interpreters, translators, negotiators or advocates for their parents and close relatives. Fulfilling these functions makes migrant children language brokers.[56, 62, 63] They translate or interpret words or sentences for their parents and relatives, but they also interpret and explain elements from the mainstream culture to their parents.[64, 65] As cultural ambassadors for their family members, migrant children help their parents function in the new culture. This new found functionality does not equate to explicit acculturation, [62, 66] rather it makes migrant children active agents in the migration and settlement processes and the capital accumulation by the family.[67–69] However, the power of children as language brokers means that parents may feel disempowered and to some extent humiliated by the fact that their children have more information and knowledge than them.[70] These changes in power dynamics challenge traditional family roles, the consequence of which is the diminished value of parents’ cultural capital[51, 55] and subsequent negative impact on family dynamics.[56]

While not a finding of this study, it is worth noting that our other studies have identified that increased power of children is also due to financial independence (e.g. not having to rely on parents for pocket money or parental approval to make a purchase) [16–18]. Upon arriving in Australia, migrant children are entitled to a number of benefits and allowances[71] that can make them financially independent. Conflicts may arise when parents and children disagree over whether youth allowance and other benefits should be subsumed into the household income, and if not, whether children on allowances or working should contribute financially to family living expenses, including the obligation to send money to relatives overseas.[72] In addition, the new found financial independence may allow children to push boundaries, assert their own independence and freedom, and claim independent decision making power and rights, which may potentially lead to family disagreements and conflicts.

## Limitations, policy recommendations and conclusion

There are few limitations of our findings worth acknowledging. Out study excluded youth aged 15–17 years and focused on youth aged 18–24 years. It is possible that youth aged 18–24 years are more resilience and have a greater sense of agency to resist parental pressures to conform to traditional values and practices and to negotiate their participation in decision making than their younger counterparts. Further studies among youth younger than 18 years are needed to validate our findings. Our data represent a description of parenting practices and behaviours among newly arrived migrant families and how they are negotiated by the migrant youth. The magnitude of these issues needed to be quantified in further quantitative studies. The fact that participants were recruited through established networks could constitute a limitation. Participants were recruited through established networks within the community meaning that only groups already formed were accessible. The extent to which these findings can be generalized is limited, given the exploratory nature of the study; further work may be required to ascertain the typicality of these findings in the wider migrant community of greater Western Sydney.

Notwithstanding this limitation, our findings suggest that migration affects youth cultural identity and the family provides an environment through which youth identity is contested. The study found that the contestation of youth identity among migrants shapes roles, relationships, values and norms inside and outside of the family. Family dynamics provide a cultural framework through which decisions and the roles of family members are determined, and a primary reference for support and negotiating post-migration experiences and challenges. Effective family interventions for migrants not only need to support parents in their role as caregivers, they should also help parents harness the increased cultural capital held by children to negotiate the threat to the family posed by children’s increased knowledge about government structures, systems and regulations. Another priority for service providers and policy makers is to put in place services that allow migrant parents to re-negotiate the parent—child relationship, notably the challenges brought about by children’s greater control over their lives and increased agency. Our findings suggest the need for an inter-generational approach to healthy family dynamics within migrant communities and in dealing with the level of complexity and sensitivity youth experience when negotiating their cultural identity. The study highlights the role of schools as sites where migrant children are socialised, and notes that parents find themselves situated outside of the school learning experience and broader school community. The centrality of schools in the process of young people negotiating their cultural identity points to a need to better link school communities to existing migrant settlement services, both to assist migrant parents build bridges into the wider community and to provide windows into their own children’s learning and acculturation experiences.
